# Clcf1/Crlf1a-mediated signaling is neuroprotective and required for Müller glia proliferation in the light-damaged zebrafish retina

**DOI:** 10.3389/fcell.2023.1142586

**Published:** 2023-02-10

**Authors:** Patrick Boyd, Leah J. Campbell, David R. Hyde

**Affiliations:** Department of Biological Sciences, Center for Stem Cells and Regenerative Medicine, and Center for Zebrafish Research, Galvin Life Sciences Building, University of Notre Dame, Notre Dame, IN, United States

**Keywords:** zebrafish, retina, regeneration, Müller glia, CLCF1, CNTFR, CRLF1

## Abstract

Zebrafish possess the innate ability to fully regenerate any neurons lost following a retinal injury. This response is mediated by Müller glia that reprogram and divide asymmetrically to produce neuronal precursor cells that differentiate into the lost neurons. However, little is understood about the early signals that induce this response. Ciliary neurotrophic factor (CNTF) was previously shown to be both neuroprotective and pro-proliferative within the zebrafish retina, however CNTF is not expressed following injury. Here we demonstrate that alternative ligands of the Ciliary neurotrophic factor receptor (CNTFR), such as Cardiotrophin-like cytokine factor 1 (Clcf1) and Cytokine receptor-like factor 1a (Crlf1a), are expressed within Müller glia of the light-damaged retina. We found that CNTFR, Clcf1, and Crlf1a are required for Müller glia proliferation in the light-damaged retina. Furthermore, intravitreal injection of CLCF1/CRLF1 protected against rod photoreceptor cell death in the light-damaged retina and induced proliferation of rod precursor cells in the undamaged retina, but not Müller glia. While rod precursor cell proliferation was previously shown to be Insulin-like growth factor 1 receptor (IGF-1R)-dependent, co-injection of IGF-1 with CLCF1/CRLF1 failed to induce further proliferation of either Müller glia or rod precursor cells. Together, these findings demonstrate that CNTFR ligands have a neuroprotective effect and are required for induction of Müller glia proliferation in the light-damaged zebrafish retina.

## 1 Introduction

The zebrafish retina possesses the ability to regenerate all retinal cell types following injury (Lahne et al., 2020). Regeneration involves Müller glia reprogramming and asymmetric division to produce neuronal precursor cells (NPCs). These NPCs continue to proliferate and eventually differentiate into the lost cell types to restore visual function (Fimbel et al., 2007; Sherpa et al., 2008; Wan & Goldman, 2016; Angueyra & Kindt, 2018; Lahne et al., 2020). Several different signalling pathways have been shown to regulate this process, including Delta/Notch (Conner et al., 2014; Wan & Goldman, 2017; Elsaeidi et al., 2018; Campbell et al., 2021), Hippo/Yap (Hoang et al., 2020; Lourenço et al., 2021), EGF (Wan et al., 2012; Ueki & Reh, 2013), and TNFα pathways (Nelson et al., 2013; Iribarne et al., 2019).

One pathway of particular interest is the ciliary neurotrophic factor receptor (CNTFR) mediated signalling pathway. CNTFR is a tripartite receptor that binds a ligand, which recruits both Il6st (Gp130) and M17 (LIFR), resulting in CNTFR phosphorylation (samuel et al., 1993; Stahl & Yancopoulos, 1994). This allows CNTFR to signal *via* the Stat3, MAPK, and PI3K pathways (Rezende et al., 2009; Leibinger et al., 2013; Askvig & Watt, 2015; Hu et al., 2020), all of which were shown to regulate the Müller glia response to injury (Kassen et al., 2009; Nelson et al., 2012; Wan et al., 2012; Wan et al., 2014). CNTFR was previously found to be expressed within zebrafish Müller glia following injury (Zhao et al., 2014). Several studies also demonstrated that CNTFR and its ligand, ciliary neurotrophic factor (CNTF), have a wide array of effects within the zebrafish retina. Knockdown of *gp130* in the damaged zebrafish retina decreased Müller glia proliferation (Zhao et al., 2014), suggesting that Gp130-mediated signalling is required for retinal regeneration. Further, intravitreal injection of CNTF was sufficient to induce zebrafish Müller glia proliferation in a Stat3-dependent manner and was neuroprotective in a MAPK pathway-dependent manner (Kassen et al., 2009; Zhao et al., 2014). CNTF treatment also resulted in reduced photoreceptor cell death in both rodents (Rhee et al., 2013; Lipinksi et al., 2015) and humans (Sieving et al., 2006; Zhang et al., 2011). Additionally, several different cell types, including Müller glia, rods, and cones, were shown to be CNTF-sensitive (Leibinger et al., 2009; Rhee et al., 2013; Li et al., 2018), with CNTF exposure stimulating significant transcriptional changes (Wang et al., 2020). Interestingly, CNTF also induced regeneration of cones in a rat model of retinal degeneration (Li et al., 2010). Finally, CNTF was also shown to confer neuroprotective effects in other areas of the central nervous system (Beurrier et al., 2010; Jeong et al., 2015).

Despite the effects CNTF exert *in vivo*, *cntf* was not shown to be expressed within the regenerating zebrafish retina, however, the alternative CNTFR ligands Cardiotrophin-like cytokine factor 1 (Clcf1) and Cytokine receptor-like factor 1a (Crlf1a), which form a ligand complex, were expressed within the retina following injury (Zhao et al., 2014). Additionally, knockdown of either *clcf1* or *crlf1a* inhibited regeneration following optic nerve crush in zebrafish (Elsaeidi et al., 2014). Despite the presence of these ligands within the retina, no in-depth studies have examined their role in Müller glia-dependent regeneration of retinal neurons. Understanding the roles of the different CNTFR ligands during zebrafish retinal damage and regeneration may guide the development of cell-based retinal therapeutics in humans.

## 2 Material and methods

### 2.1 Zebrafish husbandry

Adult *albino* and transgenic *albino; Tg[gfap:EGFP]* ^*nt11*^ zebrafish were maintained in the Center for Zebrafish Research at the University of Notre Dame Freimann Life Sciences Center as previously described (Recasens et al., 2021). Fish were maintained on a 14h/10h light-dark cycle for standard conditions. Approximately equal numbers of male and female fish were used in the study. All fish used in these experiments were 6–12 months of age and 2–3 cm in length. All animal care protocols were approved by the University of Notre Dame Animal Care and Use Committee and are compliant with the National Institutes of Health guide for the care and use of laboratory animals.

### 2.2 Light damage paradigm

Adult *albino* and *albino; Tg[gfap:EGFP]* ^*nt11*^ zebrafish were dark-adapted for 14 days and then exposed to constant intense light for up to 36 h as previously described (Garner et al., 2018). Zebrafish were euthanized by anesthetic overdose in 2-phenoxyethanol (2-PE, 1:500, Sigma-Aldrich, St. Louis, MO).

### 2.3 Drug and protein treatments

#### 2.3.1 CLCF1/CRLF1 & IGF-1

Adult *albino* and *albino; Tg[gfap:EGFP]* ^*nt11*^ zebrafish were anesthetized in 2-phenoxyethanol (2-PE, 1:1000) until unresponsive to tail pinch and intravitreally injected every 12 h with 0.5 µl of either PBS (vehicle), 1 mg/ml recombinant mouse CLCF1/CRLF1 (R&D systems; Minneapolis, MN), 1 mg/ml recombinant human IGF-1 (R&D systems; Minneapolis, MN), or a combination of both, using a 2.5 µl syringe with 33-gauge rounded needle (Hamilton; Reno, NV) and then maintained at 32°C. Eyes were collected 72 h after the initial injection (hpi). For light treatments, dark-adapted *albino* zebrafish received intravitreal injections every 12 h for 48 h, at which point the fish were exposed to constant light and received an additional injection at 12 h of light treatment. These eyes were collected at 24 h of light treatment. For EdU treatment, fish received intraperitoneal injections of 1 mg/ml EdU using a 30-gauge needle while simultaneously receiving intravitreal injections of CLCF1/CRLF1 every 12 h and eyes were collected at 72 h hpi.

#### 2.3.2 NVP-ADW742 (NVP)

*albino; Tg[gfap:EGFP]* ^*nt11*^ zebrafish were intraperitoneally injected with 50 µl of either 5% DMSO (vehicle) or 500 µM NVP (Selleck Chemical; Houston, TX) every 12 h throughout the course of either constant light treatment or intravitreal injection of CLCF1/CRLF1, using a 30-gauge needle. For light treatment experiments, the initial intraperitoneal injection of NVP corresponded with the commencement of light treatment and the final injection occurred at 24 h of light treatment, with the eyes collected at 36 h of light treatment. During CLCF1/CRLF1 treatment, intraperitoneal injections of NVP were performed simultaneously with intravitreal CLCF1/CRLF1 injections every 12 h and the eyes were collected at 72 hpi.

### 2.4 Morpholino-mediated knockdowns

Dark-adapted *albino; Tg[gfap:EGFP]* ^*nt11*^ zebrafish were intravitreally injected with 0.5 µl of either standard control, *cntfr,* a mixture of *clcf1/crlf1a,* or a combination of all 3 translation-blocking morpholinos (Gene Tools; Philomath, OR). Eyes were then electroporated as previously described (Thummel et al., 2011) before beginning constant light treatment. The following translation blocking morpholinos were used: standard control: 5′ CCT​CTT​ACC​TCA​GTT​ACA​ATT​TAT​A 3′ (Nasevicius & Ekker, 2000), *clcf1*: 5′ CCT​GAC​CAA​CTT​TCC​AGG​GAC​ACA​T 3′ (Elsaeidi et al., 2014), *cntfr:* 5′ GCG​TAA​TGC​TTC​CCT​CCT​TAT​CTT​C 3′, *crlf1a:* 5′ CAA​TAA​GCA​GAT​CAT​CTT​ACG​AGG​A 3′ (Elsaeidi et al., 2014).

### 2.5 Immunohistochemistry and EdU labelling

Immunohistochemistry was performed as previously described (Lahne et al., 2015; Lahne et al., 2021). Briefly, eyes were fixed in 9:1 ethanolic formaldehyde overnight at 4°C. The eyes were rehydrated through an ethanol gradient and incubated overnight at 4°C in 30% sucrose in PBS, followed by incubation overnight in a 2:1 mixture of tissue freezing medium (TFM):30% sucrose at 4°C. Eyes were frozen in TFM and 14 µm cryosections prepared. Sections were rehydrated in PBS for 20 min and blocked (2% normal goat serum, 2% DMSO, 1% Triton-X, 1% Tween-20 in PBS) for 1 h at room temperature. Slides were incubated with primary antibodies diluted in blocking solution overnight at room temperature. Primary antibodies used were rabbit anti-PCNA (1:2000; Abcam; Cambridge, United Kingdom; AB18197) and chicken anti-GFP (1:1000; Abcam; Cambridge, United Kingdom; AB13970). Slides were washed 3 times for 5 min in 0.1% Tween-20 in PBS (PBS-T), incubated for 1 hour with secondary antibodies and 4′,6-diamidino-2-phenylindol (DAPI; Life Technologies; Carlsbad, CA) in blocking solution at room temperature. AlexaFluor-conjugated secondary antibodies (Life Technologies; Carlsbad, CA) used were goat anti-chicken-488 (1:1000; A11039) and goat anti-rabbit-647 (1:1000; A21245). Finally, slides were washed 3 times for 5 min in PBS-T and mounted in Prolong Gold Antifade Reagent (Life Technologies; Carlsbad, CA).

For EdU labelling, slides were washed and rehydrated in PBS for 20 min, washed in 3% bovine serum albumin (BSA) in PBS for 10 min, then permeabilized by washing in 0.5% Triton X-100 (Fisher Scientific; Pittsburgh, PA) in PBS for 20 min. Slides were then labelled using the Click-iT EdU labeling kit (Life Technologies; Carlsbad, CA), per the manufacturer’s instructions, and washed in 3% BSA for 5 min before blocking and continuing with immunohistochemistry as described above.

### 2.6 *In situ* hybridization

RNA *in situ* hybridization was performed using the RNAscope Multiplex Fluorescent v2 Assay (Advanced Cell Diagnostics; Newark, CA) according to the protocol for fixed-frozen tissue sample preparation with some modifications (Campbell et al., 2021). Tissue sections were prepared as described for immunohistochemistry. Frozen sections were washed in PBS for 5 min followed by baking for 1 h at 55°C in an oven. Tissue sections were post-fixed with 4% paraformaldehyde (PFA; Sigma Aldrich; St. Louis, MO) at room temperature for 1 h. Slides were dehydrated for 5 min each in 50%, 70%, and twice in 100% ethanol and then baked for 1 h at 55°C. Sections were treated with hydrogen peroxide solution (Advanced Cell Diagnostics; Newark, CA) for 10 min at room temperature, followed by a distilled water wash and placed in boiling Target Retrieval Buffer (Advanced Cell Diagnostics; Newark, CA) for 15 min. The slides were immediately washed with distilled water, dehydrated with 100% ethanol, and dried. Slides were baked for 1 h at 55°C, during which time a hydrophobic barrier was applied to the slide (ImmEdge Hydrophobic Pen; Vector Laboratories; Burlingame, CA) and dried overnight at room temperature. Sections were treated with the Protease III solution (Advanced Cell Diagnostics; Newark, CA) for 30 min at 40°C and washed with distilled water prior to probe incubation at 40°C for 2 h. The following probes (Advanced Cell Diagnostics; Newark, CA) were used: Dr-il6st-C1 (cat no. 1108591), Dr-clcf1-C2 (cat no. 1102851), Dr-cntfr-C2 (cat no. 1102711), and Dr-crlf1a-C3 (cat no. 1102861). The 3-plex negative control probe mixture was used on a separate slide. Probe amplification with AMP1, AMP2, and AMP3 proceeded according to manufacturer instructions. Development of the HRP signal also proceeded according to manufacturer instructions with the Opal 520, Opal 570, and Opal 690 dyes (1:1000; Akoya Biosciences; Menlo Park, CA). After signal development for each probe, slides were either incubated for 5 min at room temperature with DAPI solution (Advanced Cell Diagnostics), mounted with Prolong Gold Antifade Mountant (Thermo Fisher Scientific; Waltham, MA), and coverslipped or washed in PBS-T for 5 min before proceeding with immunohistochemistry. The primary antibodies used were rabbit anti-GFP (1:1000; Abcam; Cambridge, United Kingdom; AB6556) and mouse anti-PCNA (1:1000; MilliporeSigma; Burlington, MA; P8825), followed by secondary antibodies Alexa Fluor 488-conjugated goat anti-rabbit (1:500; Thermo Fisher Scientific; Waltham, MA; A11034) and Alexa Fluor 647-conjugated goat anti-mouse (1:500; Thermo Fisher Scientific; Waltham, MA; A21245).

### 2.7 Terminal deoxynucleotidyl transferase dUTP nick end labelling (TUNEL)

Slides were washed and rehydrated in PBS and fixed in 4% PFA for 5 min at room temperature. Slides were washed 3 times for 5 min in PBS and permeabilized for 15 min in PBS-T (5% Tween-20) at room temperature. Slides were washed 3 times for 5 min in PBS and incubated for 30 min with 1:150 proteinase K (Takara Bio; Kusatsu, Shiga, Japan) in PBS at room temperature. Slides were then washed 3 times for 5 min in PBS, before the TUNEL protocol was performed as described previously (Lahne et al., 2021).

### 2.8 Image acquisition and analysis

A Nikon A1 confocal microscope, equipped with a ×40 plan-fluor oil immersion objective, was used to acquire 6.5 μm z-stacks in 0.8 μm steps, of 1024 × 1024 images of the central-dorsal region of the retina. Counts were performed manually using Fiji software throughout the 6.5 μm z-stack and normalized to a 300 μm length of the retina as previously described (Lahne et al., 2015). Statistical analyses were performed using GraphPad Prism 9 (San Diego, CA). Statistical tests used included either a Student’s *t*-test or a one-way ANOVA with a Tukey or Dunnett *post hoc* test. A *p*-value of less than 0.05 was considered significant. The mean ± SEM for each experiment is stated in the text of the appropriate Results section and the sample size (n) is stated in the appropriate figure legend.

### 2.9 Single cell RNA-seq (scRNA-seq) analysis

We used scRNA-seq data from whole light-damaged retinas that was previously published (Hoang et al., 2020). scRNA-seq analysis was performed using Seurat (Hao et al., 2021). Established clusters from the previous publication were maintained and differential expression analysis was performed comparing resting and activated Müller glia.

## 3 Results

### 3.1 CNTFR ligands Clcf1 and Crlf1a are expressed in the activated Müller glia in light-damaged retinas

**FIGURE 1:**
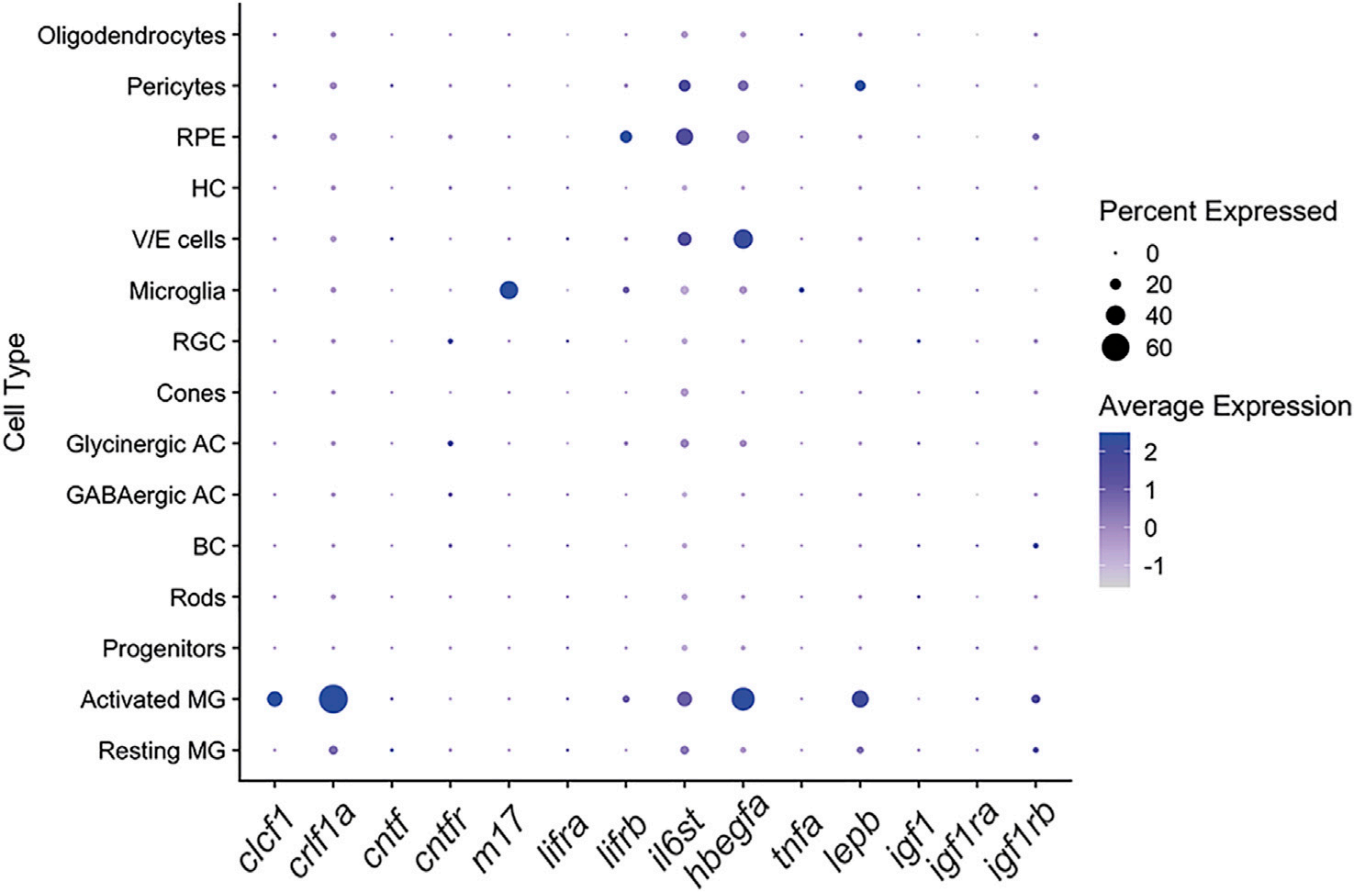
The CNTFR ligands Clcf1 and Crlf1a are expressed by Müller glia within the zebrafish retina. Dot plot showing expression of the CNTFR ligands *clcf1, crlf1a* and *cntf,* and CNTFR receptor components *cntfr, lifrb* and *il6st* in all retinal cell types in a single-cell RNA-Seq data set (Hoang et al., 2020). Expression of other pro-proliferative factors is also shown. Cells are from combined whole retina samples of 0 h, 4 h, 10 h, 20 h and 36 h of light treatment.

To investigate the spatial expression of CNTFR ligands in the light-damaged retina, we analyzed previously published scRNA-seq datasets (Hoang et al., 2020). We did not detect expression of *cntf* in any of the retinal cell types (Figure 1). In contrast, we observed expression of both *clcf1* and *crlf1a* primarily within activated Müller glia (Figure 1), suggesting that Müller glia express and secrete the Clcf1 and Crlf1a ligands in response to retinal damage. These expression patterns were similar to other key pro-proliferative factors within the zebrafish retina, such as *hbegfa* and *lepb* (Wan et al., 2012; Zhao et al., 2014). In contrast, the cytokine LIF (encoded by the *m17* gene) was expressed predominantly in microglia and not in Müller glia (Figure 1). To begin to understand how these ligands interact with cells within the damaged retina, we determined what cell types expressed any of the corresponding receptor components. We observed only low levels of *cntfr* expression in the scRNA-seq datasets, suggesting its expression was not induced during the first 36 h of retinal regeneration. Additionally, of the two LIF receptor genes encoded in zebrafish (Ogai et al., 2014), *lifra* was not significantly expressed in the retina and *lifrb* expression was upregulated in the activated Müller glia relative to the resting Müller glia (Figure 1). Finally, expression of *il6st* (encoding Gp130) was primarily limited to activated Müller glia, vascular endothelial, retinal pigmented epithelium, and pericytes, suggesting these cells are particularly sensitive to Gp130-mediated signalling. Together, these data suggest that Clcf1 and Crlf1a are released from activated Müller glia following injury and potentially interact with a variety of different cell types within the retina.

**FIGURE 2:**
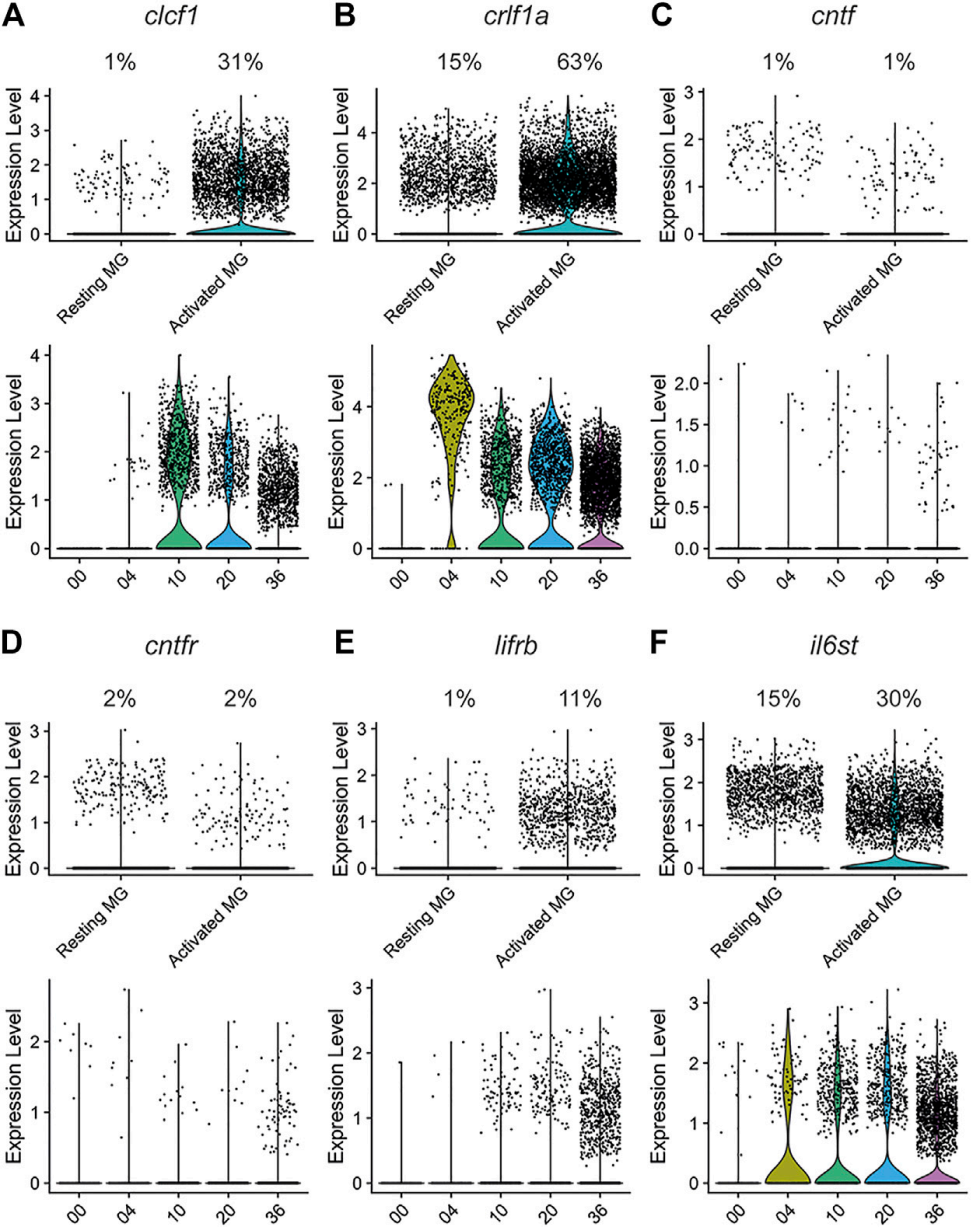
The CNTFR ligands *clcf1* and *crlf1a,* but not *cntf,* are differentially expressed by activated Müller glia during regeneration in the light-treated retina. The expression of *clcf1* **(A)**, *crlf1a* **(B)**, *cntf* **(C)**, *cntfr* **(D)**, *lifrb* **(E)**, and *il6st* **(F)** were examined in a single-cell RNA-Seq data set (Hoang et al., 2020). Upper violin plots show number of UMI expressed in individual resting and activated Müller glia. Percentage of Müller glia in each cluster expressing the gene of interest is shown above the plot. Lower violin plots show number of UMI expressed across time in activated Müller glia.

We further explored the expression of *clcf1* and *crlf1a*, and several associated genes, in both resting and activated Müller glia, as well as across the damage time course within the activated Müller glia population. Both *clcf1* (*p* < 1.00 × 10^−200^) and *crlf1a* (*p* < 1.00 × 10^−200^) exhibited a significant increase in expression within the activated population of Müller glia (Figures 2A,B, respectively). In addition, *clcf1* and *crlf1a* expression were upregulated by 10 h and 4 h of light damage (Figures 2A,B, respectively). This suggests that Müller glia upregulates the expression of both genes prior to photoreceptor cell death and they both may be required for either neuroprotection or proliferation. Little expression of *cntf* was observed in either resting or activated Müller glia (Figure 2C), indicating that Clcf1 and Crlf1a are the primary CNTFR ligands expressed within the retina. In contrast, *clcf1* expression was significantly increased (*p* = 7.40 × 10^−183^) in activated Müller glia in the light-treated mouse retina (Supplementary Figure S1A), while *crlf1* expression was significantly decreased (*p* = 0.03) in activated Müller glia relative to resting Müller glia (Supplementary Figure S1B). Similar to zebrafish, we did not detect expression of *cntf* in either mouse Müller glia population following light treatment (Supplementary Figure S1C).

In the zebrafish retina, expression of the receptor components *cntfr* (Figure 2D) and *lifra* (Figure 1) was relatively low in both Müller glia populations. In contrast, *lifrb* expression (Figure 2E) was significantly higher in activated Müller glia (*p* = 6.00 × 10^−153^) and increased from 10 through 36 h of light treatment. Similarly, *il6st* significantly increased in expression in activated Müller glia (*p* = 2.80 × 10^−71^) from 4 through at least 36 h of light treatment (Figure 2F). This rapid induction and sustained expression of *il6st* suggests that Gp130-mediated signalling may have both neuroprotective and pro-proliferative effects. The expression differences seen in the receptor genes may be due to Gp130 forming a receptor complex in both a CNTFR- and LIFR-independent manner, and therefore is likely required for additional processes (White & Stevens, 2011). Interestingly, *Cntfr* expression was observed in resting mouse Müller glia, however *Cntfr* was significantly downregulated in activated Müller glia (Supplementary Figure S1D; *p* = 7.11 × 10^−24^). No change was observed in *Lifr* expression (Supplementary Figure S1E), but *Il6st* expression was significantly increased within activated Müller glia (Supplementary Figure S1F; *p* = 3.80 × 10^−11^).

**FIGURE 3:**
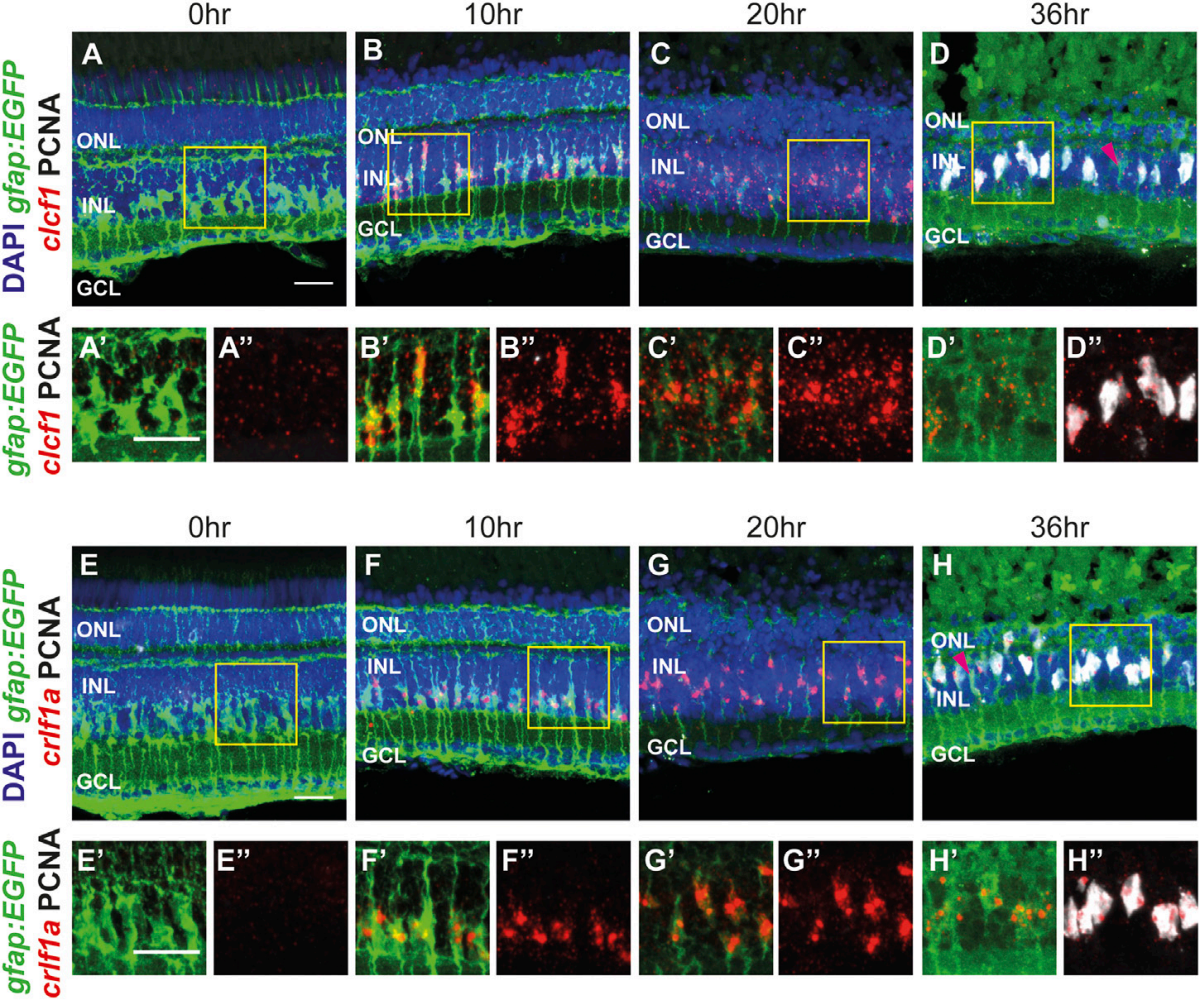
The CNTFR ligands *clcf1* and *crlf1a* are detected within Müller glia following injury, consistent with scRNA-seq data. **(A**–H")Maximum projection images of dark-adapted *albino; Tg[gfap:EGFP]* ^*nt11*^ zebrafish that were light-treated for up to 36 h, with *in situ* hybridization performed utilizing either *clcf1* **(A**–D") or *crlf1a* **(E**–H") probes. Sections were also labeled for GFP and PCNA to assess proliferating Müller glia and nuclei were counterstained with DAPI. Yellow boxes in **(A-H)** represent areas chosen for greater magnification (in A'-H' and A"-H"). Magenta arrowheads **(D, H)** show resting Müller glia. ONL, outer nuclear layer, INL, inner nuclear layer, GCL, ganglion cell layer. Scale bar represents 20 µm.

To confirm that *clcf1* and *crlf1a* were expressed in Müller glia, we performed *in situ* hybridization using the RNAscope system. Dark-adapted *albino; Tg[gfap:EGFP]* ^*nt11*^ fish were placed in intense light treatment for either 0 (undamaged), 10, 20, or 36 h before eyes were collected and retinal sections were labeled for either *clcf1 or crlf1a,* GFP, and PCNA. A broad and low level of *clcf1* expression was detected at 0 h (Figures 3A–A’’). However, *clcf1* expression increased and colocalized with GFP-expressing Müller glia (*Tg(gfap:EGFP)*) from 10 through 20 h (Figures 3B–C’’). By 36 h of light treatment, the *clcf1* signal appeared weaker and more dispersed throughout the retina (Figures 3D–D’’). However, *clcf1* puncta were still observed within proliferating Müller glia (Figures 3D, D’’), suggesting that *clcf1* was expressed in Müller glia that would enter the cell cycle. Little expression of *crlf1a* was observed at 0 h (Figures 3E–E’’). However, by 10 and 20 h of light treatment, *crlf1a* was observed within Müller glia (Figures 3F–G’’) and persisted within proliferating Müller glia through 36 h of light treatment (Figures 3H–H’’). Furthermore, *clcf1* and *crlf1a* were observed within the same Müller glia at 36 h of light treatment (Supplementary Figures S2A, S2B). Additionally, PCNA-negative Müller glia also did not express either *clcf1* or *crlf1a* (Figures 3D,H), which likely represent resting Müller glia. These expression patterns closely resemble the expression time course identified by scRNA-seq and revealed that *clcf1* and *crlf1a* are highly upregulated within Müller glia early in the damage paradigm, before photoreceptor cell death.

We also examined the expression patterns of the *cntfr* and *il6st* receptors*.* Expression of *cntfr* was observed broadly throughout the retina from 0 to 36 h of light treatment (Supplementary Figures S3A–S3D’’) with no obvious changes in expression level or pattern, which is consistent with the scRNA-seq data (Figure 1). A similar expression pattern was observed for *il6st* (Supplementary Figures S3E–S3H’’). This data suggests that cells within each nuclear layer may respond to the release of Clcf1 and Crlf1a from Müller glia following injury.

### 3.2 Intravitreal injection of recombinant CLCF1/CRLF1 protects against photoreceptor cell death during light treatment

**FIGURE 4:**
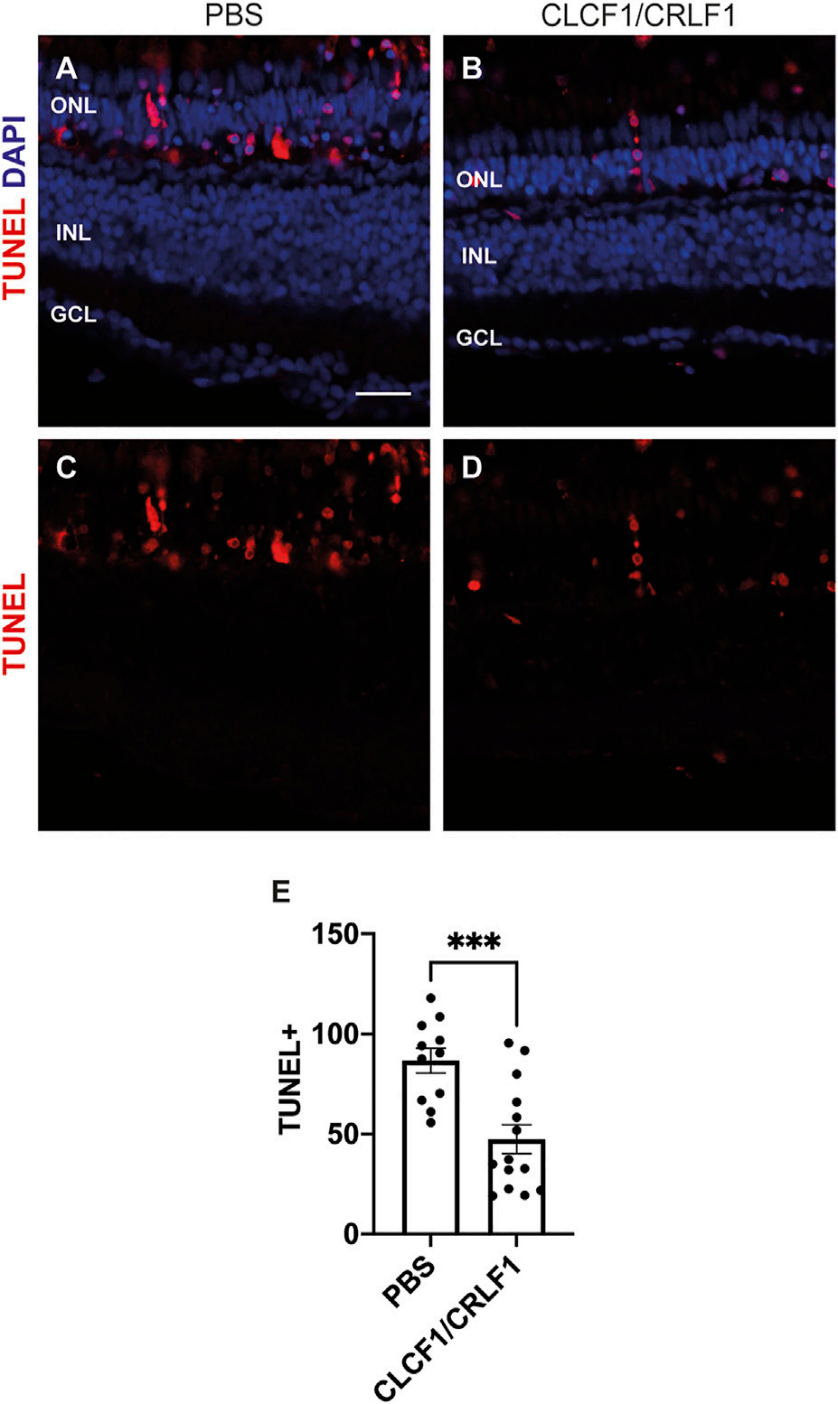
Intravitreal injection of CLCF1/CRLF1 protects against photoreceptor cell death during light treatment. **(A–D)** Single z-plane confocal images from dark-adapted *albino* zebrafish that were light-treated for 24 h while being intravitreally injected with either PBS **(A, C)** or CLCF1/CRLF1 **(B, D)**. TUNEL assay was performed to assess cell death, and nuclei were counterstained with DAPI. **(E)** Quantification showing significant decrease in the number of TUNEL-positive nuclei within the ONL of CLCF1/CRLF1-treated retinas. Student’s *t*-test, *p* = 0.0005, n ≥ 11. Mean ± SEM, ***p < 0.001. ONL, outer nuclear layer, INL, inner nuclear layer, GCL, ganglion cell layer. Scale bar represents 20 µm.

Previous studies determined that CNTF is neuroprotective within both the zebrafish and mammalian retinas (Kassen et al., 2009; Aslam et al., 2013). We examined whether Clcf1 and Crlf1a, which are alternative ligands for the CNTF receptor, can provide similar neuroprotective benefits. We intravitreally injected *albino* zebrafish with either PBS (vehicle) or 1 mg/ml CLCF1/CRLF1 every 12 h until 48 h after the initial injection, at which point fish were placed in constant intense light. They received a further injection at 12 h of constant light and eyes were collected 12 h later (24 h light treatment). To quantify cell death, we performed the TUNEL assay on retinal sections (Figures 4A–D). We observed a significant decrease in the number of TUNEL-positive ONL cells in Clcf1/Crlf1a-co-injected retinas relative to vehicle control (Figure 4E; PBS: 86.68 ± 6.19, CLCF1/CRLF1: 47.34 ± 7.18, *p* = 0.0005). This demonstrates that *clcf1/crlf1a* co-expression by zebrafish Müller glia may be neuroprotective in the light-damaged retina.

### 3.3 CNTFR and the associated ligands Clcf1 and Crlf1a are required for Müller glia proliferation in the light-treated retina

**FIGURE 5:**
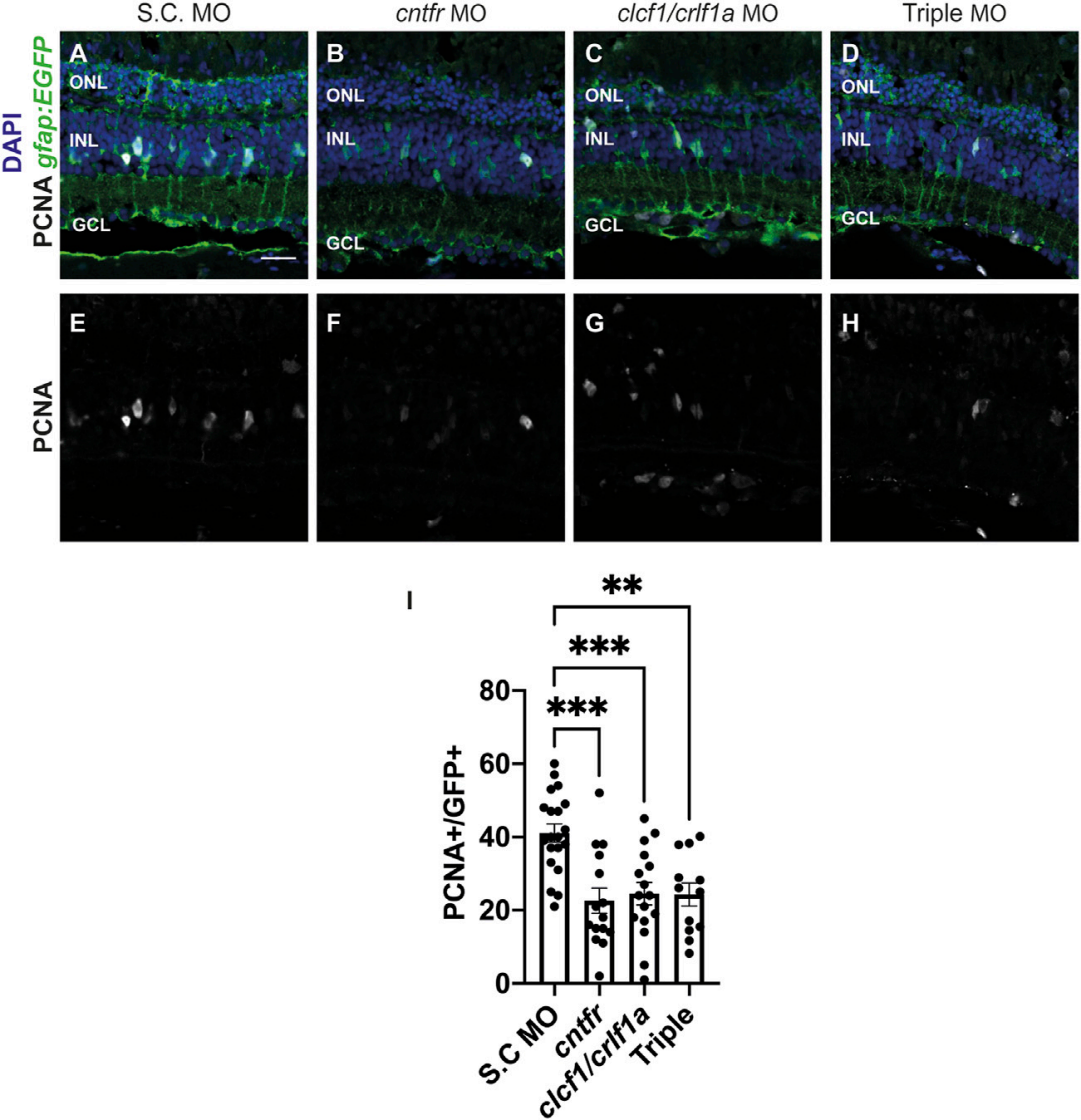
Knockdown of CNTFR and Clcf1/Crlf1a reduces Müller glia proliferation. **(A–H)** Single confocal images from dark-adapted *albino; Tg[gfap:EGFP]* ^nt11^ zebrafish which were light-treated for 36 h after electroporation of either S.C. **(A)**, *cntfr* **(B),** *clcf1/crlf1a* morpholinos **(C),** or a combination of all three *cntfr/clcf1/crlf1a* (Triple) **(D)**. Sections were labeled for GFP and PCNA to assess proliferating Müller glia and nuclei were counterstained with DAPI **(E, F, G, H)**. **(I)** Quantification showing significant decrease in the number of PCNA-positive Müller glia within the INL in *cntfr* and *clcf1/crlf1a* electroporated retinas. One-way ANOVA with Dunnett *post hoc*, *cntfr* vs. S.C. *p* < 0.0001, *clcf1/crlf1a* vs. S.C. *p* = 0.0003, Triple vs. S.C. *p* = 0.0072. n ≥ 12. Mean ± SEM, **p < 0.001, ***p < 0.001, ****p < 0.0001. ONL, outer nuclear layer, INL, inner nuclear layer, GCL, ganglion cell layer. Scale bar represents 20 µm.

We examined whether CNTFR, Clcf1, or Crlf1a contribute to Müller glia proliferation following light-induced photoreceptor cell death. Dark-adapted *albino; Tg[gfap:EGFP]* ^*nt11*^ fish were electroporated with either standard control morpholino (S.C. MO) (Figures 5A,E), which does not correspond to any known sequence in the zebrafish genome (Nasevicius & Ekker, 2000), *cntfr* morpholino (Figures 5B,F), a combination of the *clcf1* and *crlf1a* MOs (Figures 5C,G), or a mixture of all three (Triple; Figures 5D,H) before being placed in constant light treatment. Retinas were collected at 36 h of constant light and labelled for GFP and PCNA to quantify proliferating Müller glia. As compared to the S.C. MO group (Figure 5I; S.C.: 36.59 ± 2.15 PCNA-positive Müller glia), a significant decrease in the number of proliferating Müller glia was observed in the *cntfr* MO group (Figure 5I; *cntfr*: 17.82 ± 2.83, *p* < 0.0001), the *clcf1/crlf1* MO group (Figure 5I; *clcf1/crlf1a:* 21.62 ± 2.84, *p* = 0.0003), and the Triple MO groups (Figure 5I; Triple: 24.31 ± 3.16, *p* = 0.0072). There was no significant difference between the *cntfr* MO, *clcf1/crlf1* MO, and the Triple MO groups. This suggests that both the receptor and the Clcf1/Crlf1a ligands are required for Müller glia proliferation following light-induced damage. Additionally, this result suggested that while Clcf1/Crlf1a co-expression may be neuroprotective in the light-damaged retina (Figure 4), it is not necessary.

We next examined whether the reduction in Müller glia proliferation was a result of reduced cell death in the morphants by electroporating *albino* zebrafish with either S.C. (Supplementary Figures S4A, S4E), *cntfr* (Supplementary Figures S4B, S4F), *clcf1/crlf1a* (Supplementary Figures S4C, S4G)*,* or all three morpholinos (Triple MO, Supplementary Figures S4D, S4H) and collecting the retinas at 24 h of light treatment. The TUNEL assay was performed to assess cell death (Supplementary Figures S4A–S4H). No significant differences in the number of TUNEL-positive cells were observed between any treatment group relative to S.C. (Supplementary Figure S4I; S.C.: 109.9 ± 8.75 TUNEL-positive cells, *cntfr*: 112.5 ± 4.19 *p* = 0.98, *clcf1/crlf1a*: 114.7 ± 7.46 *p* = 0.92, Triple: 112.4 ± 5.12 *p* = 0.99), suggesting that the reduced number of proliferating Müller glia in either the *cntfr, clcf1*/*crlf1a* or the Triple morphants was not due to decreased cell death, but rather their requirement for stimulating Müller glia proliferation, with Clf1/Crlf1a likely acting as ligands for CNTFR in the light-treated retina.

### 3.4 Intravitreal injection of recombinant CLCF1/CRLF1 induces rod precursor cell proliferation

**FIGURE 6:**
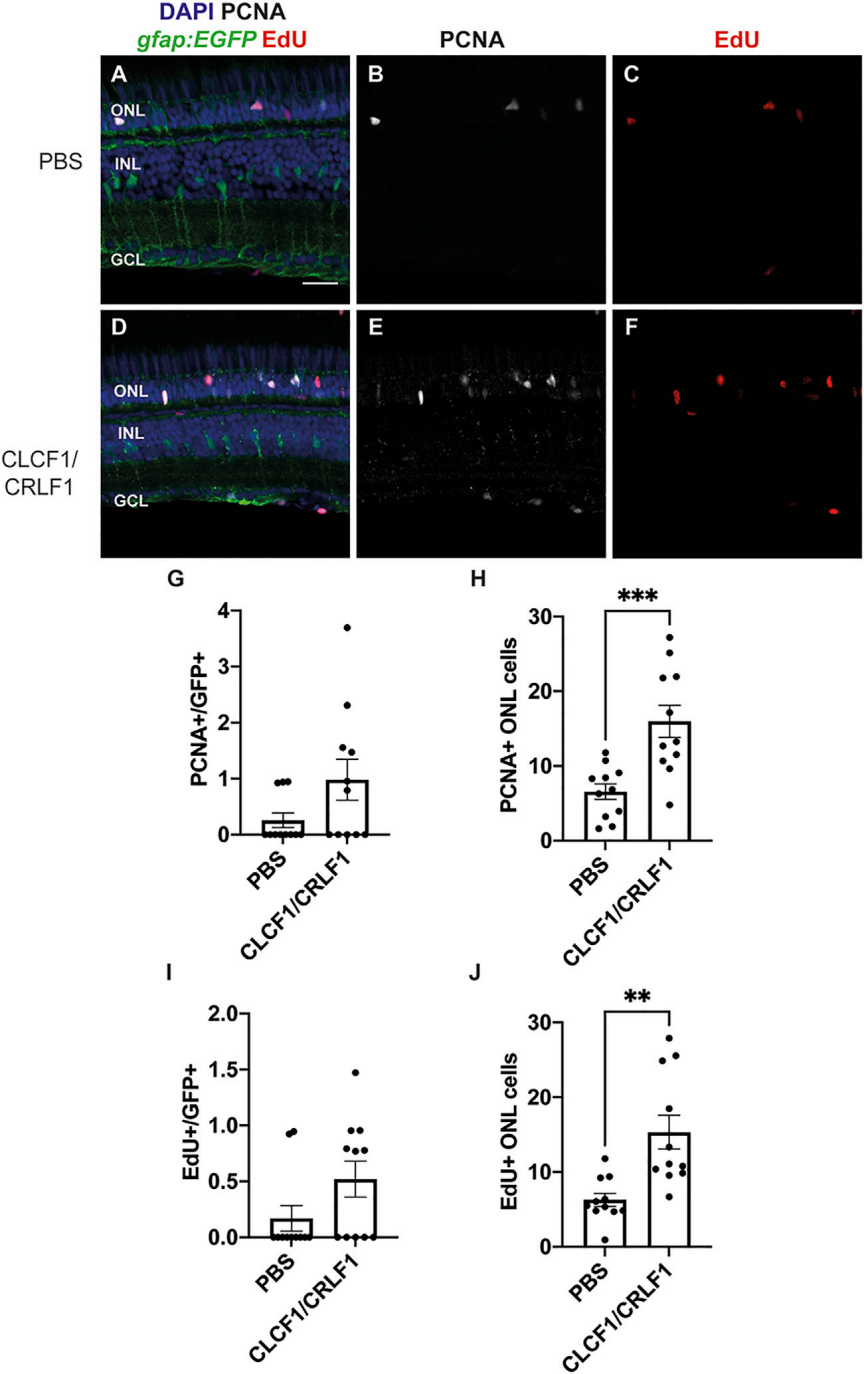
Intravitreal injection of recombinant mouse CLCF1/CRLF1 induces rod precursor cell proliferation. **(A–F)** Single confocal images from *albino; Tg[gfap:EGFP]* ^nt11^ zebrafish which were intravitreally injected every 12 h with either PBS **(A)** or CLCF1/CRLF1 **(D)** for 72 h. Sections were labeled for GFP, PCNA, and EdU to assess proliferating Müller glia and nuclei were counterstained with DAPI **(B, C, E, F)**. **(G)** Quantification showing no significant change in the number of PCNA-positive Müller glia within the INL of CLCF1/CRLF1-injected retinas. Student’s t-test, *p* = 0.08. **(H)** Quantification showing significant increase in the number of PCNA-positive cells within the ONL of CLCF1/CRLF1-injected retinas. Student’s *t*-test, *p* = 0.0008. **(I)** Quantification showing no significant difference in the number of EdU-positive Müller glia within the INL of CLCF1/CRLF1-injected retinas. Student’s *t*-test, *p* = 0.09. **(J)** Quantification showing significant increase in the number of EdU-positive cells within the ONL of CLCF1/CRLF1-injected retinas. Student’s *t*-test, *p* = 0.0014. n ≥ 11. Mean ± SEM, **p < 0.01, ***p < 0.001. ONL, outer nuclear layer, INL, inner nuclear layer, GCL, ganglion cell layer. Scale bar represents 20 µm.

Since Clcf1/Crlf1a signalling is required for Müller glia proliferation, we examined whether co-injection of mouse CLCF1/CRLF1 was sufficient to induce zebrafish Müller glia proliferation. Either PBS (vehicle, Figures 6A–C) or 1 mg/ml of both mouse CLCF1 and CRFL1 (Figures 6D–F) was intravitreally injected into *albino; Tg[gfap:EGFP]* ^*nt11*^ fish along with an interperitoneal injection of the thymidine analogue EdU to label proliferating cells. Injections occurred every 12 h until 72 h after the initial injection, at which time eyes were collected and retinal sections were labelled for GFP, PCNA, and EdU to assess proliferating Müller glia and EdU-labelled cells. We observed no significant difference in the number of PCNA-positive Müller glia (Figure 6G; PBS: 0.26 ± 0.13, CLCF1/CRFL1: 0.98 ± 0.36, *p* = 0.08) or EdU-positive Müller glia (Figure 6I; PBS: 0.17 ± 0.11, CLCF1/CRFL1: 0.52 ± 0.16, *p* = 0.09) between vehicle and CLCF1/CRFL1-treated groups. This suggests that CLCF1/CRFL1-mediated signalling was insufficient to induce Müller glia proliferation. However, we observed a significant increase in the number of PCNA-positive ONL cells (Figure 6H; PBS: 6.56 ± 1.06, CLCF1/CRFL1: 16.0 ± 2.16, *p* = 0.0008) and EdU-positive ONL cells (Figure 6J; PBS: 6.23 ± 0.88, CLCF1/CRLF1: 15.3 ± 2.27, *p* = 0.0014) in the CLCF1/CRFL1-injected retinas relative to vehicle control. These proliferating ONL cells may represent rod precursor cells that produce rod photoreceptors during persistent retinal neurogenesis (Morris et al., 2008; Wilson et al., 2016) and CLCF1/CRFL1 treatment may be sufficient to induce further rod precursor cell proliferation without increased Müller glia proliferation.

### 3.5 CLCF1/CRFL1-induced rod precursor cell proliferation is IGF-1R dependent

**FIGURE 7:**
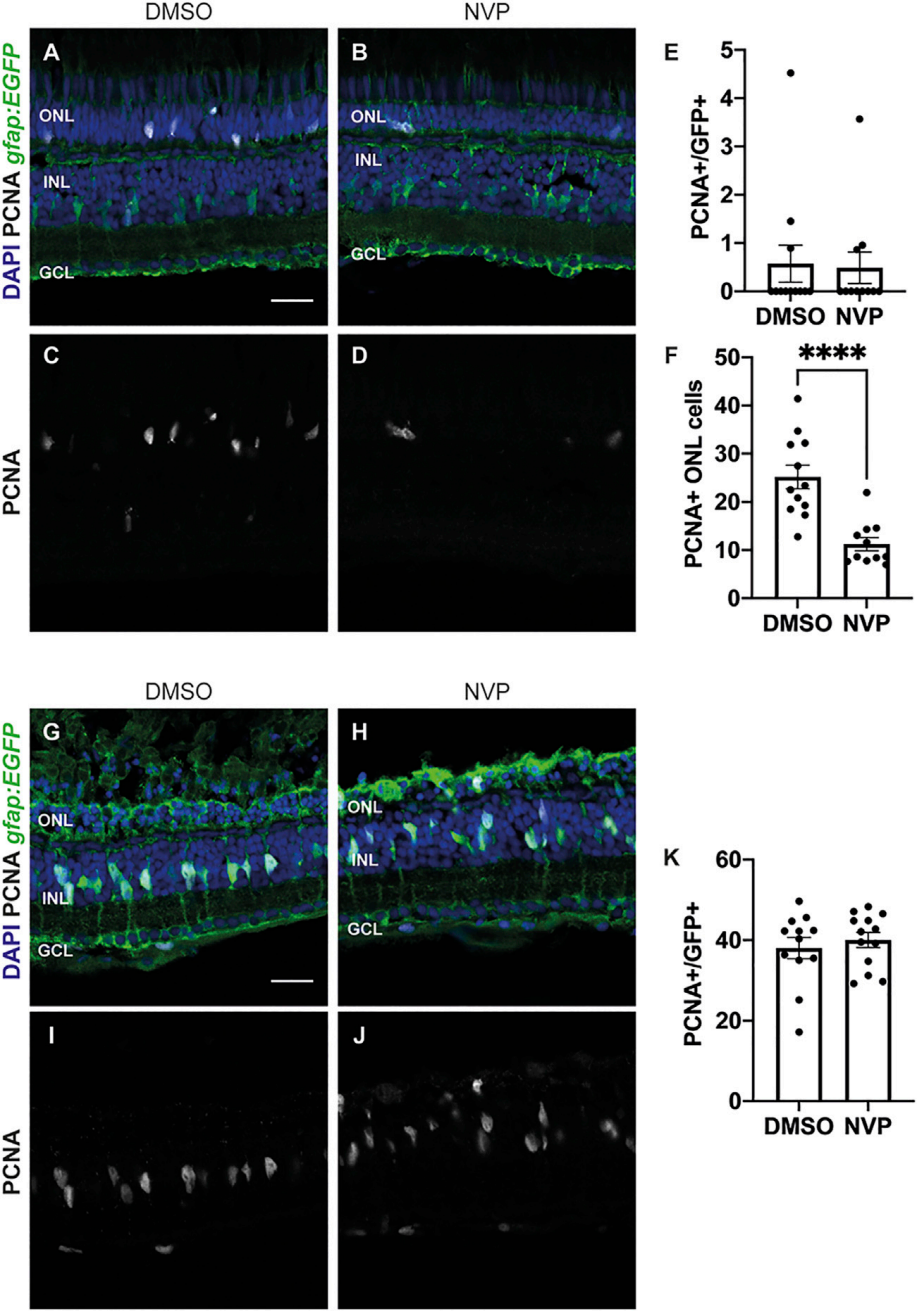
CLCF/CRLF1 induces rod precursor cell proliferation in an IGF-1R dependent manner. **(A–D)** Single confocal images from *albino; Tg[gfap:EGFP]* ^*nt11*^ zebrafish which were intravitreally injected every 12 h with CLCF1/CRLF1 and simultaneously receiving injections of either DMSO (vehicle) **(A)** or the IGF-1R inhibitor NVP **(B)** for 72 h. Sections were labeled for GFP and PCNA to assess proliferating Müller glia and nuclei were counterstained with DAPI **(C, D)**. **(E)** Quantification showing no significant change in the number of PCNA-positive Müller glia within the INL of NVP-treated fish. Student’s *t*-test, *p* = 0.87, n ≥ 11. **(F)** Quantification showing significant decrease in the number of PCNA-positive cells within the ONL of NVP-treated fish. Student’s *t*-test, *p* = 0.0001, n ≥ 11. **(G–J)** Single confocal images from dark-adapted *albino; Tg[gfap:EGFP]* ^*nt11*^ zebrafish which were light-treated for 36 h while receiving injections of either DMSO (vehicle) **(G)** or the IGF-1R inhibitor NVP **(H)**. Sections were labeled for GFP and PCNA to assess proliferating Müller glia and nuclei were counterstained with DAPI **(I, J)**. **(K)** Quantification showing no significant change in the number of PCNA-positive Müller glia between DMSO and NVP-treated fish. Student’s t-test, *p* = 0.54, n ≥ 12. Mean ± SEM, ****p < 0.0001. ONL, outer nuclear layer, INL, inner nuclear layer, GCL, ganglion cell layer. Scale bar represents 20 µm.

Previous studies showed that rod precursor cell proliferation requires Insulin-like growth factor 1 receptor 1 (IGF-1R) (Zygar et al., 2005; Lahne et al., 2019). To determine if CLCF1/CRFL1-induced rod precursor cell proliferation is IGF-1R-dependent, *albino; Tg[gfap:EGFP]* ^*nt11*^ zebrafish were intravitreally injected with 1 mg/ml CLCF1/CRFL1 and intraperitoneally injected with either DMSO (vehicle; Figures 7A,C) or the IGF-1R inhibitor NVP (Figures 7B,D). Fish received both injections every 12 h. At 72 h after the initial injection, retinas were isolated, sectioned, and labelled for GFP and PCNA to assess Müller glia proliferation. We observed no significant difference in the number of PCNA-positive Müller glia between the DMSO and NVP-treated groups (Figure 7E; DMSO: 0.57 ± 0.38, NVP: 0.49 ± 0.33, *p* = 0.87). However, there was a significant decrease in the number of proliferating rod precursor cells in the NVP-treated group relative to vehicle control (Figure 7F; DMSO: 25.17 ± 2.43, NVP: 11.23 ± 1.36, *p* < 0.0001). This suggests that CLCF1/CRFL1-induced rod precursor cell proliferation was IGF-1R dependent. To confirm that increased rod precursor cell proliferation was not a product of increased Müller glia proliferation, dark-adapted *albino; Tg[gfap:EGFP]* ^*nt11*^ zebrafish were placed in constant intense light treatment, while also receiving intraperitoneal injections of either DMSO (Figures 7G,I) or NVP (Figures 7H,J) beginning at the start of the light treatment and every 12 h thereafter. Eyes were collected after 36 h of constant light treatment and sections were labelled for GFP and PCNA to assess Müller glia proliferation. We did not detect a significant change in the number of PCNA-positive Müller glia between treatment groups (Figure 7K; DMSO: 38.04 ± 2.63, NVP: 40.03 ± 1.88, *p* = 0.54), suggesting that inhibiting IGF-1R does not affect Müller glia proliferation. This is consistent with scRNA-seq data that revealed little expression of either *igf1* or the associated receptor components (*igf1ra* and *igf1rb*) in Müller glia in the light-damaged retina (Figure 1). Together these data suggest that CLCF1/CRFL1 induces rod precursor proliferation in an IGF-1R-dependent manner.

### 3.6 Intravitreal co-injection of IGF-1 and Clcf1/Crlf1a is not sufficient to induce Müller glia proliferation or additional rod precursor proliferation

**FIGURE 8:**
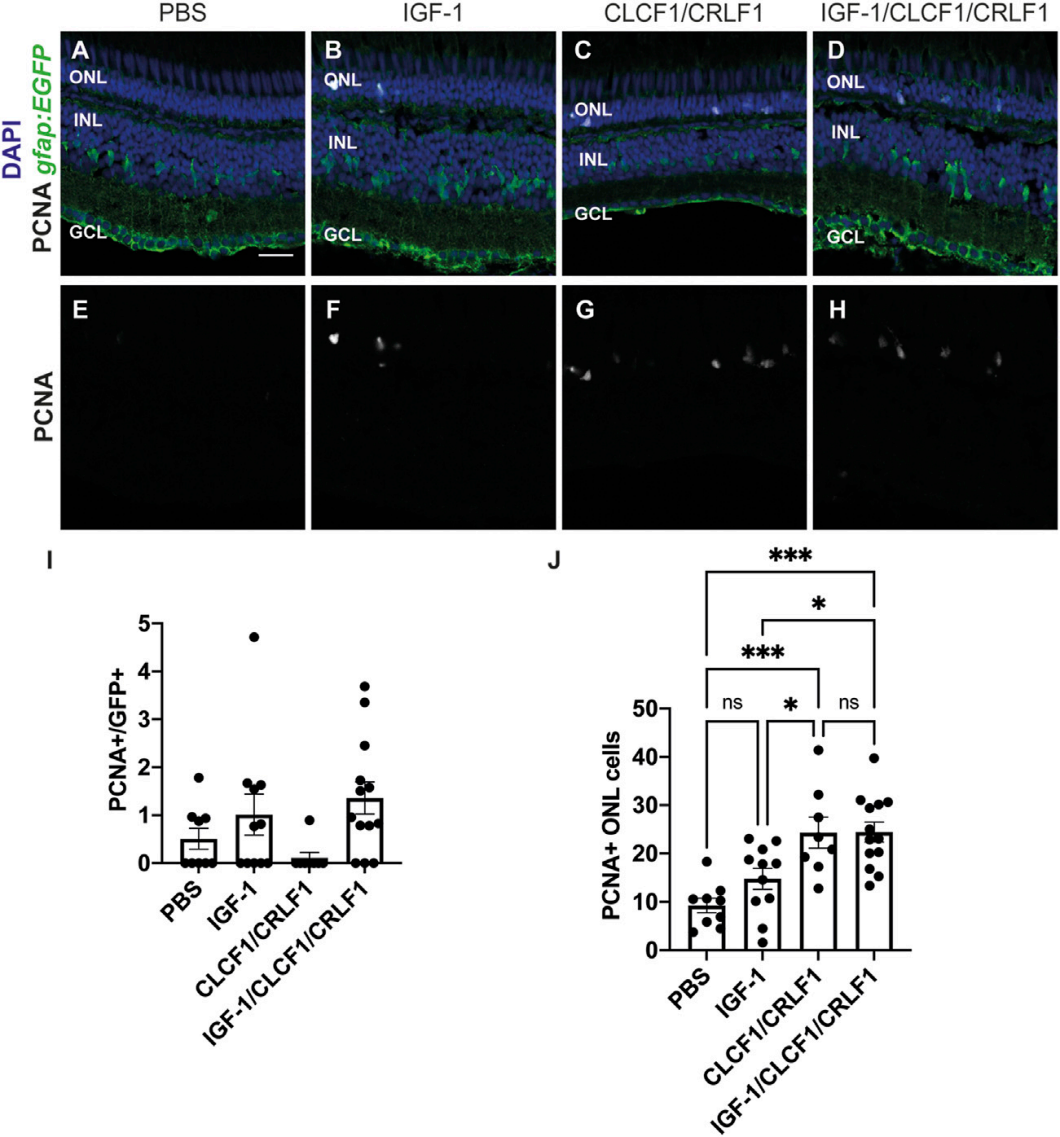
Co-injection of CLCF/CRLF and IGF-1 does not induce additional proliferation. **(A–D)** Single confocal images from *albino; Tg[gfap:EGFP]* ^nt11^ zebrafish which were intravitreally injected every 12 h with either PBS **(A)**, IGF-1 **(B)**, CLCF1/CRLF1 **(C),** or a combination of IGF-1 and Clcf1/Crlf1a **(D)** for 72 h. Sections were labeled for GFP and PCNA to assess proliferating Müller glia and nuclei were counterstained with DAPI **(E, F, G, H)**. **(I)** Quantification showing no change in the number of PCNA-positive Müller glia within the INL of IGF-1 and IGF-1/CLCF1/CRLF1-injected retinas. **(J)** Quantification showing significant difference in the number of PCNA-positive cells within the ONL of IGF-1 and IGF-1/CLCF1/CRLF1-injected retinas. One-way ANOVA with Tukey *post hoc*, IGF-1/CLCF1/CRLF1 vs. PBS *p* = 0.0001, CLCF1/CRLF1 vs. PBS *p* = 0.0007, IGF-1 vs. CLCF1/CRLF1 *p* = 0.0362, IGF-1/CLCF1/CRLF1 vs. IGF-1 *p* = 0.0127. n ≥ 8. Mean ± SEM, *p < 0.05,***p < 0.001. ONL, outer nuclear layer, INL, inner nuclear layer, GCL, ganglion cell layer. Scale bar represents 20 µm.

Since Clcf1/Crlf1a-dependent rod precursor cell proliferation is IGF-1R-dependent, we examined if co-injection of IGF-1 and Clcf1/Crlf1a was sufficient to induce further proliferation. Previous studies demonstrated that IGF-1 synergizes with FGF2 to induce significant Müller glia proliferation within the zebrafish retina (Wan et al., 2014), therefore we hypothesized that the combination of IGF-1 and Clcf1/Crlf1a would induce a similar response in the absence of retinal damage. Either PBS (vehicle) (Figures 8A,E), IGF-1 (Figures 8B,F), Clcf1/Crlf1a (Figures 8C,G), or a combination of IGF-1 and Clcf1/Crlf1a (Figures 8D,H) were intravitreally injected into *albino;Tg[gfap:EGFP]* ^*nt11*^ fish every 12 h. At 72 h after the initial injection, eyes were collected, sectioned, and labelled for GFP and PCNA to assess proliferation (Figures 8A–H). We observed no significant difference in the number of PCNA-positive Müller glia between any groups (Figure 8I; PBS: 0.51 ± 0.22, IGF-1: 1.01 ± 0.43, Clcf1/Crlf1a: 0.11 ± 0.11, IGF-1/Clcf1/Crlf1a: 1.36 ± 0.33), suggesting that co-injection of IGF-1 and Clcf1/Crlf1a was not sufficient to induce Müller glia proliferation in the absence of retinal damage. Furthermore, no significant difference was observed in the number of PCNA-positive rod precursor cells between PBS and IGF-1-injected groups (Figure 8J; PBS: 9.24 ± 1.52, IGF-1: 14.76 ± 2.18, *p* = 0.34), indicating that IGF-1 was not sufficient to induce rod precursor cell proliferation. However, there was a significant difference in the number of proliferating rod precursor cells between the PBS and Clcf1/Crlf1a-treated groups (Clcf1/Crlf1a: 24.32 ± 3.23, *p* = 0.0007), and PBS and IGF-1/Clcf1/Crlf1a-treated groups (IGF-1/Clcf1/Crlf1a: 24.44 ± 2.09, *p* = 0.0001). Additionally, a significant increase in the number of proliferating rod precursor cells was observed between Clcf1/Crlf1a and IGF-1-treated groups (*p* = 0.036), and the IGF-1/Clcf1/Crlf1a and IGF-1 groups (*p* = 0.013), but there was no significant difference between the Clcf1/Crlf1a and IGF-1/Clcf1/Crlf1a-treated groups (*p* = 0.99). This suggests that although Clcf1/Crlf1a-induced rod precursor cell proliferation is IGF-1R-dependent, addition of IGF-1 does not induce further proliferation of rod precursor cells.

## 4 Discussion

This is the first demonstration that the Ciliary neurotrophic factor receptor (CNTFR) ligands Cardiotrophin-like cytokine factor 1 (Clcf1) and Cytokine receptor-like factor 1a (Crlf1a) are required for retinal regeneration. We investigated expression of these ligands and their corresponding receptor components within the damaged retina and showed that Clcf1 and Crlf1a are neuroprotective, are required for Müller glia proliferation, and can induce rod precursor proliferation in an IGF-1R-dependent manner.

Previously, it was demonstrated that both *clcf1* and *crlf1a* are expressed in Müller glia-derived precursor cells at 96 h post injury (Zhao et al., 2014), however no in-depth analyses have fully investigated the spatio-temporal expression or function of either ligand. Our scRNA-seq data demonstrate that both *clcf1 a*nd *crlf1a* are expressed rapidly following retinal damage with Müller glia expressing these ligands as early as 4 h post injury and maintaining expression until at least 36 h post injury. This expression pattern was further confirmed by *in situ* hybridization, which demonstrates co-expression of *clcf1* and *crfl1a* in proliferating MG, indicating that these ligands are expressed specifically in activated MG. In contrast, we observed little expression of *cntf*, which was previously shown to be neuroprotective in both mammalian (Tao et al., 2002; Sieving et al., 2006; Azadi et al., 2007; Rhee et al., 2013) and zebrafish models of retinal damage (Kassen et al., 2009) and can induce Müller glia proliferation within zebrafish (Kassen et al., 2009; Zhao et al., 2014). This suggests that Clcf1 and Crlf1a are the primary CNTFR ligands expressed within the regenerating retina and may have similar effects on the retina.

Similar to the effects of CNTF (Kassen et al., 2009), intravitreal injection of CLCF1/CRLF1 significantly reduces photoreceptor cell death during light treatment, indicating that Clcf1 and Crlf1a may play a neuroprotective role following damage, which is further supported by the expression of these ligands prior to the occurrence of cell death. Additionally, knockdown of *clcf1* and *crlf1a* significantly reduced Müller glia proliferation following light treatment, however intravitreal injection of CLCF1/CRLF1 was insufficient to induce Müller glia proliferation in the undamaged retina. Therefore, Clcf1 and Crlf1a are necessary, but not sufficient, for the induction of Müller glia proliferation. Clcf1 and Crlf1a have also been shown to be required for optic nerve regeneration (Elsaeidi et al., 2014), suggesting a general role for these ligands in neuronal regeneration. The contrast in Müller glia proliferation between CNTF and CLCF1/CRLF1-treated retinas may be due to differences in downstream actions of these ligands. Mutations in CRLF1 or CLCF1 have been linked to cold-induced sweating syndrome, however mutations in CNTF do not induce similar effects (Murakami et al., 2019), although both have been demonstrated to induce expression and phosphorylation of Stat3 (Kassen et al., 2009; Nahlé et al., 2019; Rezende et al., 2009; Savin et al., 2015 which has been shown to be essential in Müller glia for retinal regeneration (Nelson et al., 2012).

Despite the inability to increase Müller glia proliferation, intravitreal injection of CLCF1/CRLF1 did induce a significant increase in rod precursor cell proliferation. A similar increase in rod precursor cell proliferation was observed following intravitreal CNTF injection (Kassen et al., 2009). It has previously been demonstrated that proliferation of rod precursor cells is IGF-1R1R-dependent (Lahne et al., 2019). We demonstrated that CLCF1/CRLF1-induced rod precursor cell proliferation is IGF-1R-dependent, although Müller glia proliferation following injury is not. It has previously been shown that IGF-1 can synergize with FGF2 to induced Müller glia proliferation, and that *igfra* is required for Müller glia proliferation following injury (Wan et al., 2014), however co-injection of IGF-1 with CLCF1/CRLF1 failed to induce additional Müller glia or rod precursor cell proliferation. Therefore, the factors that may synergize with Clcf1 and Crlf1a to induce Müller glia proliferation following injury remain unclear.

To better understand how these ligands operate within the zebrafish retina, we also investigated expression of the receptor complex components. NPCs have previously been shown to express *cntfr* at 96 h post injury (Zhao et al., 2014). Within scRNA-seq datasets we observed little expression of *cntfr*, however *in situ* hybridization demonstrated that *cntfr* is expressed throughout the retina at all timepoints studied. Knockdown of *cntfr* also demonstrated that CNTFR is required for Müller glia proliferation. We observed a significant increase in expression of *lifrb* and *il6st* within activated Müller glia, and *in situ* hybridization confirmed that *il6st* is expressed throughout the retina at all timepoints. These findings suggest that Müller glia, and likely most cell types of the retina, are sensitive to CNTFR ligands. This is further supported by transcriptional changes in multiple cell types following CNTF exposure in the mouse retina (Wang et al., 2020).

Together this data suggests a model in which Müller glia respond to injury by producing the CNTFR ligands Clcf1 and Crlf1a, which play a dual role within the regenerating retina showing both neuroprotective and pro-proliferative properties. Based upon expression of receptor components, Clcf1 and Crlf1a can signal to most cell types. It has previously been shown within the mouse retina that the neuroprotective effects of CNTF require GP130 within Müller glia (Rhee et al., 2013), therefore changes induced in Müller glia in response to CNTFR ligands may provide the observed neuroprotective effects, rather than direct CNTFR-mediated signalling within damaged cell types. CNTF has also previously been shown to interact with microglia (Kahn et al., 1995; Baek et al., 2018), which are known to be required during retinal regeneration (Conedera et al., 2019; Mitchell et al., 2019). Therefore, the potential interaction of Clcf1 and Crlf1a with microglia within the damaged retina may be one way in which these two cell types interact.

## Data Availability

The datasets presented in this study can be found in online repositories. All scRNA-seq data and source codes are available at GitHub https://github.com/jiewwwang/Single-cell-retinalregeneration. The scRNA-seq data can be queried interactively at https://proteinpaint.stjude.org/F/2019.retina.scRNA.html.

## Abbreviations

DAPI, 4′,6-diamidino-2-phenylindol; DMSO, dimethyl sulfoxide; EdU, 5-ethynyl-2′-deoxyuridine; EGFP, enhanced green fluorescent protein; GCL, ganglion cell layer; INL, inner nuclear layer; NPC, neuronal precursor cell; NVP, NVP-ADW742; ONL, outer nuclear layer; PBS, phosphate buffered saline; PCNA, proliferating cell nuclear antigen; TUNEL, terminal deoxynucleotidyl transferase dUTP nick end labelling.
