# Supplementary figures and images for: Clcf1/Crlf1a-mediated signaling is neuroprotective and required for Müller glia proliferation in the light-damaged zebrafish retina
Source: Front Cell Dev Biol. 2023 Feb 10;11:1142586. doi: 10.3389/fcell.2023.1142586 (PMC9950120; doi:10.3389/fcell.2023.1142586)

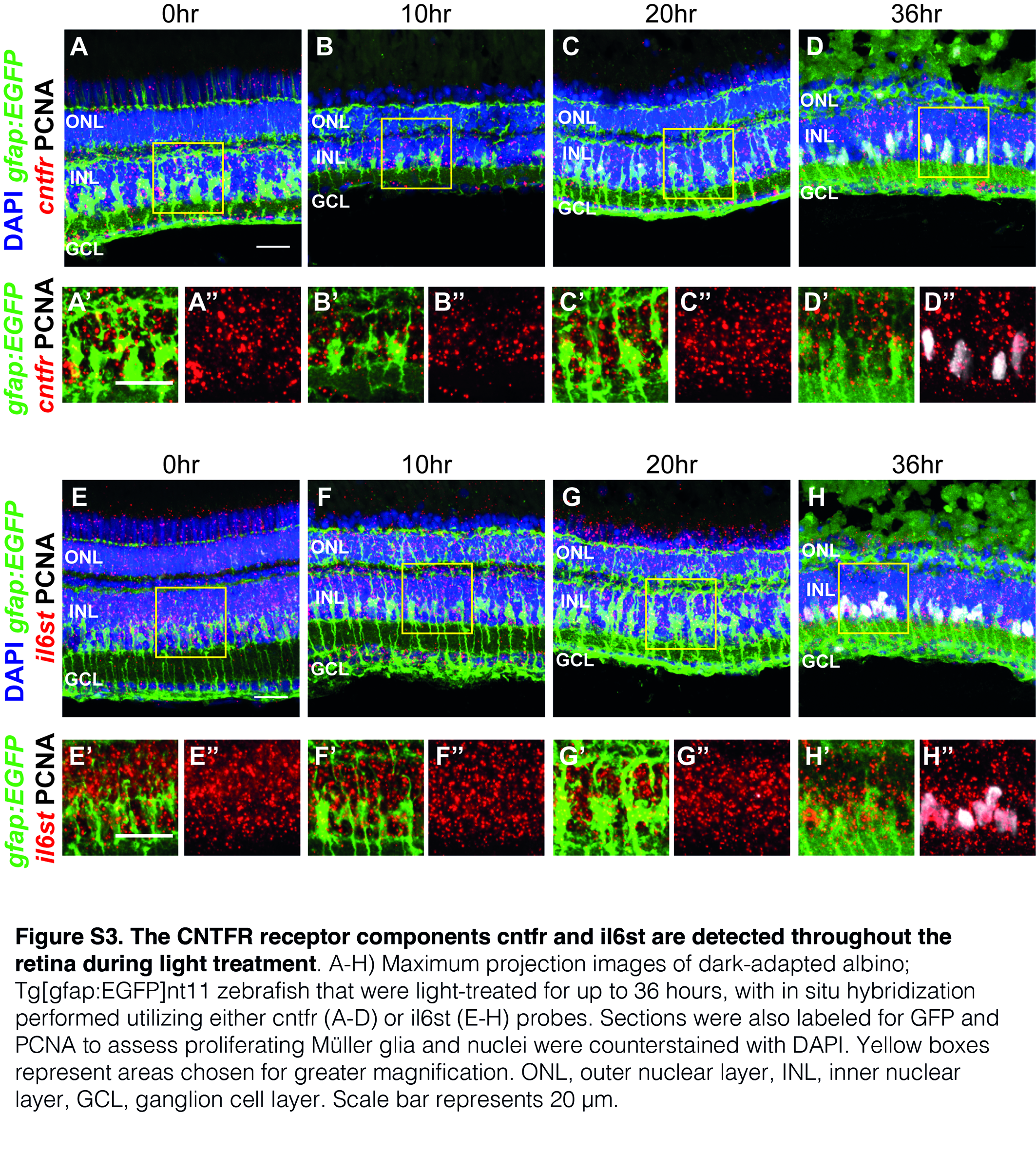

Supplement: Supplementary file 1 [file Image3.TIF]

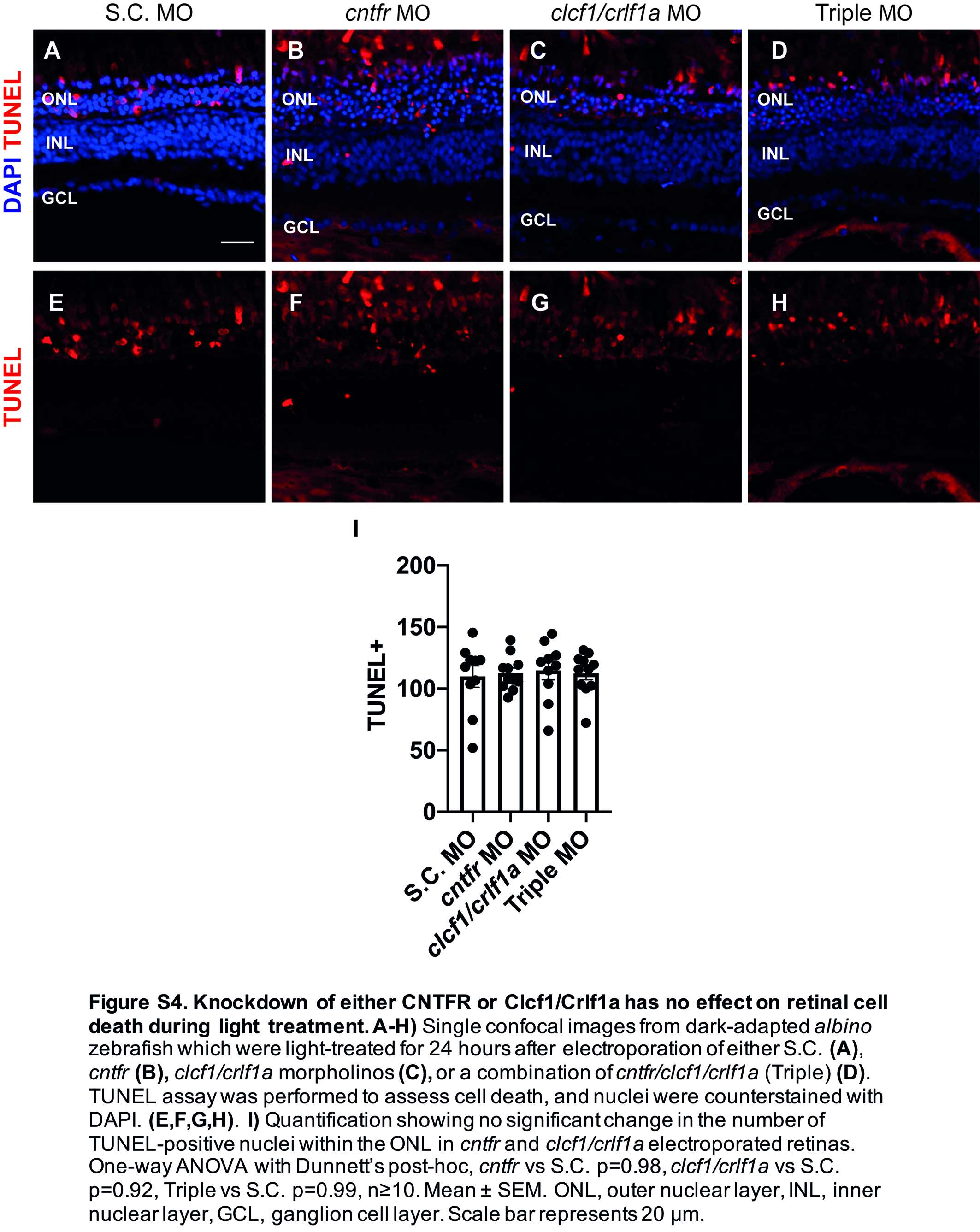

Supplement: Supplementary file 2 [file Image4.TIF]

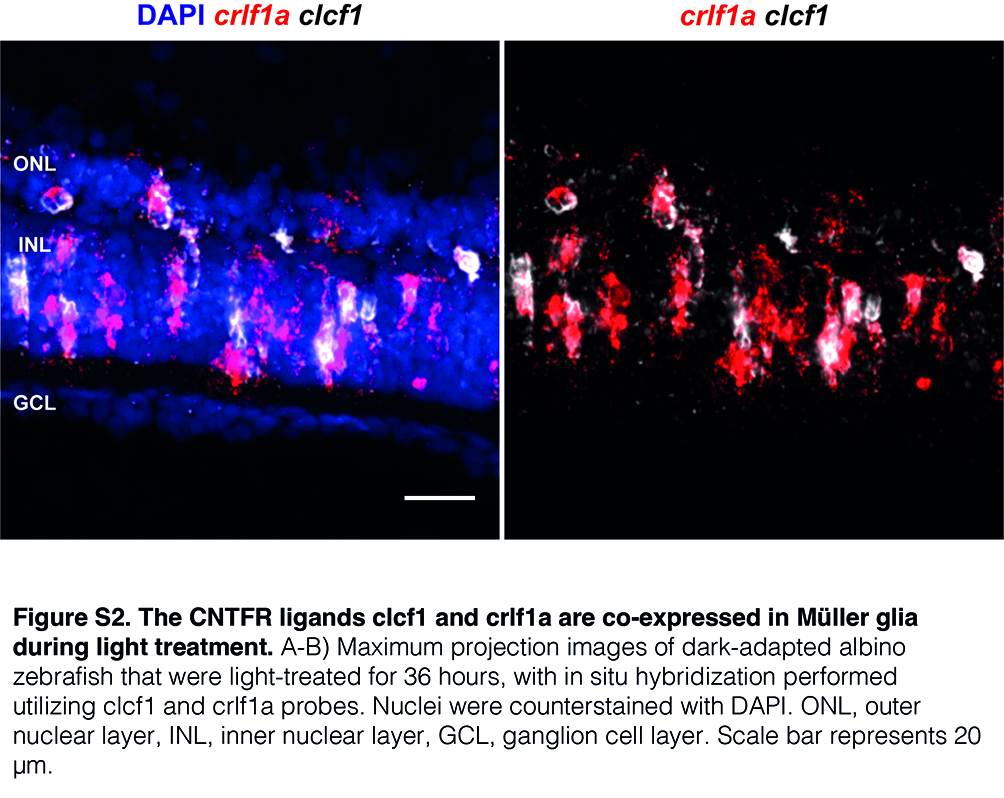

Supplement: Supplementary file 3 [file Image2.TIF]

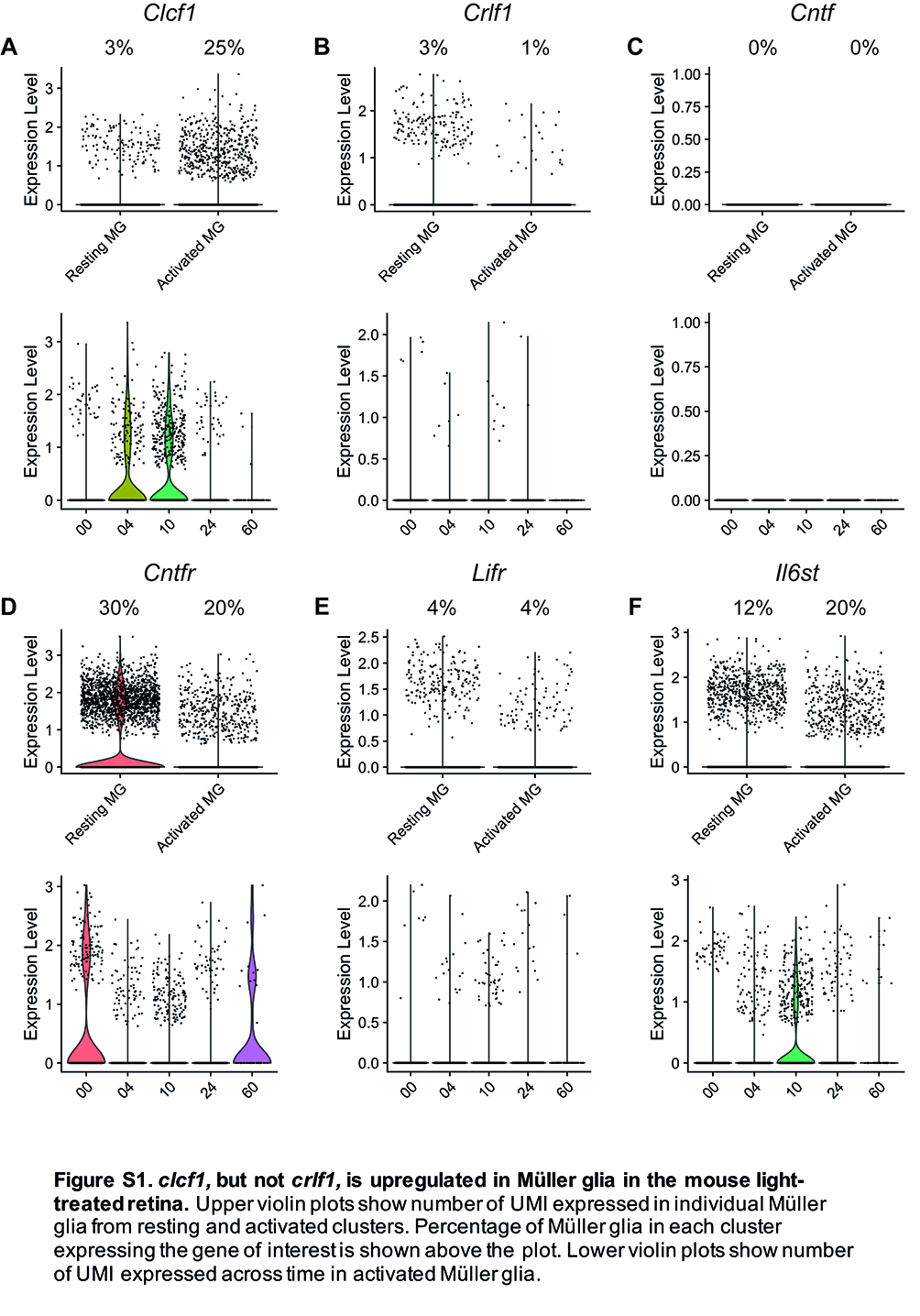

Supplement: Supplementary file 4 [file Image1.TIF]
